# Supplementary material for: End Sequence Analysis Toolkit (ESAT) expands the extractable information from single-cell RNA-seq data
Source: Genome Res. 2016 Oct;26(10):1397–410. doi: 10.1101/gr.207902.116 (PMC5052061; doi:10.1101/gr.207902.116)
Supplement: Supplemental Material [file supp_gr.207902.116_Supp_Materials.tar.gz › ESAT-esat_v0.1/doc/html/classbroad_1_1pda_1_1annotation_1_1_locus.html]

ESAT: broad.pda.annotation.Locus Class Reference


ESAT


- Main Page
- Related Pages
- Packages
- Classes
- Files

- Class List
- Class Index
- Class Hierarchy
- Class Members

All Classes Namespaces Files Functions Variables Enumerator Pages

- broad
- pda
- annotation
- Locus

Public Member Functions |
Static Public Member Functions |
List of all members

broad.pda.annotation.Locus Class Reference

|  |  |
| --- | --- |
| Public Member Functions | |
|  | Locus (GeneWithIsoforms g) |
|  | |
| void | addIsoform (GeneWithIsoforms iso, String set) |
|  | |
| GeneWithIsoforms | getReferenceTranscript () |
|  | |
| HashMap< String, Integer > | getNumIsoformsPerSet () |
|  | |
| HashMap< String, Double > | getMaxScorePerSet () |
|  | |
| HashMap< String, Integer > | getIsComaptibleWithRefPerSet () |
|  | |
| HashMap< String, Integer > | getIsPartiallyComaptibleWithRefPerSet () |
|  | |
| int | getIsComaptibleBetweenSubsets (ArrayList< String > set1Lst, ArrayList< String > set2Lst, boolean isFully) |
|  | |
| int | getIsComaptibleBetweenAny2Sets (ArrayList< String > setLst, boolean isFully) |
|  | |
| int | getIsComaptibleBetweenSubsets (ArrayList< String > set1Lst, ArrayList< String > set2Lst, BEDFileParser uniqIsoBed) |
|  | |
| int | getIsComaptibleBetweenAny2Sets (ArrayList< String > set3Lst, BEDFileParser uniqIsoBed) |
|  | |
| Gene | getExonUnion () |
|  | |
| double[] | getDistanceTo3primeNeighbor (BEDFileParser geneSet) throws IOException |
|  | |
| double[] | getDistanceTo5primeNeighbor (BEDFileParser geneSet) throws IOException |
|  | |
| double[] | distanceToOppositeStrand5primeNeighbor (BEDFileParser geneSet) throws IOException |
|  | |
| double | getMaxScoreAcrossAllIso () |
|  | |
| void | updateIsoformsNameWithLocusName () |
|  | |
| Collection< Gene > | getSetIsoforms (String setName) |
|  | |
| Collection< Gene > | getAllIsoforms (String excludeSet) |
|  | |
| Collection< Gene > | getComaptibleBetweenAny2Sets (ArrayList< String > setLst, boolean isFully) |
|  | |
| Collection< Gene > | getUnqComaptibleBetweenAny2Sets (ArrayList< String > setLst, Collection<?extends Gene > unqIsoSet, boolean isFully) |
|  | |
| Collection< Gene > | getUnqComaptibleBetweenSubsets (ArrayList< String > set1Lst, ArrayList< String > set2Lst, Collection< Gene > unqIsoSet, boolean isFully) |
|  | |
| Collection< Gene > | getComaptibleIsoforms (Gene g, boolean isFully) |
|  | |
| Gene | getLongestIntornChainIso (String refSet) |
|  | |
| Gene | getMerged () |
|  | |
| List< Gene > | getAllMerged () |
|  | |
| int | numOfOverlapSets (ArrayList< String > setLst) |
|  | |
| double | getMaxPctCovered () |
|  | |
| double | getMaxExonsCovered () |
|  | |
| double | getMaxPctGenomeCovered () |
|  | |
| HashMap< Gene, ArrayList  < Double > > | pctOverlapWithRef () |
|  | |
| int | isTerminiCoverageBias () |
|  | |
| Collection<?extends Gene > | getAllIsoforms () |
|  | |
| Integer | getNumberOfIsoforms () |
|  | |
| Collection<?extends Gene > | selectRandIsoSubset (Integer maxIsoPerLoci) |
|  | |

|  |  |
| --- | --- |
| Static Public Member Functions | |
| static Collection< Gene > | SelectLongestIntronChainCandidate (Collection< Gene > betweenSubsetsIsoSet) |
|  | |
| static boolean | isFullyComaptible (Gene iso1, Gene iso2, boolean stringent) |
|  | |

## Constructor & Destructor Documentation

|  |  |  |  |  |  |
| --- | --- | --- | --- | --- | --- |
| broad.pda.annotation.Locus.Locus | ( | GeneWithIsoforms | *g* | ) |  |

## Member Function Documentation

|  |  |  |  |
| --- | --- | --- | --- |
| void broad.pda.annotation.Locus.addIsoform | ( | GeneWithIsoforms | *iso*, |
|  |  | String | *set* |
|  | ) |  |  |

|  |  |  |  |  |  |
| --- | --- | --- | --- | --- | --- |
| double [] broad.pda.annotation.Locus.distanceToOppositeStrand5primeNeighbor | ( | BEDFileParser | *geneSet* | ) | throws IOException |

|  |  |  |  |  |  |
| --- | --- | --- | --- | --- | --- |
| Collection<Gene> broad.pda.annotation.Locus.getAllIsoforms | ( | String | *excludeSet* | ) |  |

|  |  |  |  |  |
| --- | --- | --- | --- | --- |
| Collection<? extends Gene> broad.pda.annotation.Locus.getAllIsoforms | ( |  | ) |  |

Here is the caller graph for this function:

|  |  |  |  |  |
| --- | --- | --- | --- | --- |
| List<Gene> broad.pda.annotation.Locus.getAllMerged | ( |  | ) |  |

Here is the caller graph for this function:

|  |  |  |  |
| --- | --- | --- | --- |
| Collection<Gene> broad.pda.annotation.Locus.getComaptibleBetweenAny2Sets | ( | ArrayList< String > | *setLst*, |
|  |  | boolean | *isFully* |
|  | ) |  |  |

Here is the call graph for this function:

|  |  |  |  |
| --- | --- | --- | --- |
| Collection<Gene> broad.pda.annotation.Locus.getComaptibleIsoforms | ( | Gene | *g*, |
|  |  | boolean | *isFully* |
|  | ) |  |  |

Here is the call graph for this function:

|  |  |  |  |  |  |
| --- | --- | --- | --- | --- | --- |
| double [] broad.pda.annotation.Locus.getDistanceTo3primeNeighbor | ( | BEDFileParser | *geneSet* | ) | throws IOException |

Here is the call graph for this function:

|  |  |  |  |  |  |
| --- | --- | --- | --- | --- | --- |
| double [] broad.pda.annotation.Locus.getDistanceTo5primeNeighbor | ( | BEDFileParser | *geneSet* | ) | throws IOException |

Here is the call graph for this function:

|  |  |  |  |  |
| --- | --- | --- | --- | --- |
| Gene broad.pda.annotation.Locus.getExonUnion | ( |  | ) |  |

Here is the caller graph for this function:

|  |  |  |  |
| --- | --- | --- | --- |
| int broad.pda.annotation.Locus.getIsComaptibleBetweenAny2Sets | ( | ArrayList< String > | *setLst*, |
|  |  | boolean | *isFully* |
|  | ) |  |  |

Here is the call graph for this function:

|  |  |  |  |
| --- | --- | --- | --- |
| int broad.pda.annotation.Locus.getIsComaptibleBetweenAny2Sets | ( | ArrayList< String > | *set3Lst*, |
|  |  | BEDFileParser | *uniqIsoBed* |
|  | ) |  |  |

Here is the call graph for this function:

|  |  |  |  |
| --- | --- | --- | --- |
| int broad.pda.annotation.Locus.getIsComaptibleBetweenSubsets | ( | ArrayList< String > | *set1Lst*, |
|  |  | ArrayList< String > | *set2Lst*, |
|  |  | boolean | *isFully* |
|  | ) |  |  |

Here is the call graph for this function:

|  |  |  |  |
| --- | --- | --- | --- |
| int broad.pda.annotation.Locus.getIsComaptibleBetweenSubsets | ( | ArrayList< String > | *set1Lst*, |
|  |  | ArrayList< String > | *set2Lst*, |
|  |  | BEDFileParser | *uniqIsoBed* |
|  | ) |  |  |

Here is the call graph for this function:

|  |  |  |  |  |
| --- | --- | --- | --- | --- |
| HashMap<String,Integer> broad.pda.annotation.Locus.getIsComaptibleWithRefPerSet | ( |  | ) |  |

|  |  |  |  |  |
| --- | --- | --- | --- | --- |
| HashMap<String,Integer> broad.pda.annotation.Locus.getIsPartiallyComaptibleWithRefPerSet | ( |  | ) |  |

|  |  |  |  |  |  |
| --- | --- | --- | --- | --- | --- |
| Gene broad.pda.annotation.Locus.getLongestIntornChainIso | ( | String | *refSet* | ) |  |

|  |  |  |  |  |
| --- | --- | --- | --- | --- |
| double broad.pda.annotation.Locus.getMaxExonsCovered | ( |  | ) |  |

|  |  |  |  |  |
| --- | --- | --- | --- | --- |
| double broad.pda.annotation.Locus.getMaxPctCovered | ( |  | ) |  |

|  |  |  |  |  |
| --- | --- | --- | --- | --- |
| double broad.pda.annotation.Locus.getMaxPctGenomeCovered | ( |  | ) |  |

|  |  |  |  |  |
| --- | --- | --- | --- | --- |
| double broad.pda.annotation.Locus.getMaxScoreAcrossAllIso | ( |  | ) |  |

|  |  |  |  |  |
| --- | --- | --- | --- | --- |
| HashMap<String,Double> broad.pda.annotation.Locus.getMaxScorePerSet | ( |  | ) |  |

|  |  |  |  |  |
| --- | --- | --- | --- | --- |
| Gene broad.pda.annotation.Locus.getMerged | ( |  | ) |  |

Here is the call graph for this function:

|  |  |  |  |  |
| --- | --- | --- | --- | --- |
| Integer broad.pda.annotation.Locus.getNumberOfIsoforms | ( |  | ) |  |

|  |  |  |  |  |
| --- | --- | --- | --- | --- |
| HashMap<String,Integer> broad.pda.annotation.Locus.getNumIsoformsPerSet | ( |  | ) |  |

|  |  |  |  |  |
| --- | --- | --- | --- | --- |
| GeneWithIsoforms broad.pda.annotation.Locus.getReferenceTranscript | ( |  | ) |  |

|  |  |  |  |  |  |
| --- | --- | --- | --- | --- | --- |
| Collection<Gene> broad.pda.annotation.Locus.getSetIsoforms | ( | String | *setName* | ) |  |

|  |  |  |  |
| --- | --- | --- | --- |
| Collection<Gene> broad.pda.annotation.Locus.getUnqComaptibleBetweenAny2Sets | ( | ArrayList< String > | *setLst*, |
|  |  | Collection<?extends Gene > | *unqIsoSet*, |
|  |  | boolean | *isFully* |
|  | ) |  |  |

|  |  |  |  |
| --- | --- | --- | --- |
| Collection<Gene> broad.pda.annotation.Locus.getUnqComaptibleBetweenSubsets | ( | ArrayList< String > | *set1Lst*, |
|  |  | ArrayList< String > | *set2Lst*, |
|  |  | Collection< Gene > | *unqIsoSet*, |
|  |  | boolean | *isFully* |
|  | ) |  |  |

|  |  |  |  |
| --- | --- | --- | --- |
| static boolean broad.pda.annotation.Locus.isFullyComaptible | ( | Gene | *iso1*, |
|  |  | Gene | *iso2*, |
|  |  | boolean | *stringent* |
|  | ) |  |  |

static

Here is the caller graph for this function:

|  |  |  |  |  |
| --- | --- | --- | --- | --- |
| int broad.pda.annotation.Locus.isTerminiCoverageBias | ( |  | ) |  |

|  |  |  |  |  |  |
| --- | --- | --- | --- | --- | --- |
| int broad.pda.annotation.Locus.numOfOverlapSets | ( | ArrayList< String > | *setLst* | ) |  |

|  |  |  |  |  |
| --- | --- | --- | --- | --- |
| HashMap<Gene, ArrayList<Double> > broad.pda.annotation.Locus.pctOverlapWithRef | ( |  | ) |  |

Here is the call graph for this function:

|  |  |  |  |  |  |
| --- | --- | --- | --- | --- | --- |
| static Collection<Gene> broad.pda.annotation.Locus.SelectLongestIntronChainCandidate | ( | Collection< Gene > | *betweenSubsetsIsoSet* | ) |  |

static

Here is the call graph for this function:

|  |  |  |  |  |  |
| --- | --- | --- | --- | --- | --- |
| Collection<? extends Gene> broad.pda.annotation.Locus.selectRandIsoSubset | ( | Integer | *maxIsoPerLoci* | ) |  |

Here is the call graph for this function:

|  |  |  |  |  |
| --- | --- | --- | --- | --- |
| void broad.pda.annotation.Locus.updateIsoformsNameWithLocusName | ( |  | ) |  |

Here is the call graph for this function:

---

The documentation for this class was generated from the following file:

- java/broad/pda/annotation/Locus.java


---

Generated on Fri Aug 15 2014 15:10:58 for ESAT by  

 1.8.7
